# Psychological Profiles and Clinical Clusters of Patients Diagnosed With Functional Neurological Disorder

**DOI:** 10.3389/fneur.2020.580267

**Published:** 2020-10-15

**Authors:** Paul Pun, Julanne Frater, Megan Broughton, Rian Dob, Alexander Lehn

**Affiliations:** ^1^Emotional Health Unit, Mater Misericordiae Health Services Brisbane Ltd, Brisbane, QLD, Australia; ^2^Brisbane Clinical Neuroscience Centre, Brisbane, QLD, Australia; ^3^Map and Mind Psychology, Brisbane, QLD, Australia; ^4^Department of Neurology, Princess Alexandra Hospital, Woolloongabba, QLD, Australia; ^5^The University of Queensland Princess Alexandra Hospital Clinical School, Woolloongabba, QLD, Australia

**Keywords:** functional neurological disorders, conversion disorder, psychological profiles, neuropsychiatry, non-epileptic seizures

## Abstract

Our understanding about underlying mechanisms leading to Functional Neurological Disorders (FND) has changed in recent years. While in the past these disorders were presumed to be solely due to psychological issues we know now that their development is dependent on complex interactions between biological, psychological and social factors. We present an analysis of clinical presentations and psychological profiles of patients who were seen in our FND outpatient clinic over 3 years. We aim to review the prevalence of common symptoms in the patients seen within our clinic, and to identify any common psychological or psychiatric profiles that differentiated these symptom groups. This may help to elucidate underlying mechanisms leading to the development of functional symptoms and identify the predisposing, triggering and perpetuation factors.

## Introduction

Patients suffering from Functional Neurological Disorders (FNDs) can present with a variety of symptoms: Common presentations include weakness or functional movement disorders, such as tremors and spasms. Dissociative attacks cause periods of altered or loss of consciousness and/or abnormal movements, which can look like epileptic seizures or syncopes. The diagnosis of FNDs relies on positive signs demonstrating internal inconsistency and/or incongruity with other recognized neurological conditions. Approximately 6% of neurology outpatient contacts receive a diagnosis of FND (1) although as many as 30% have some form of medically unexplained neurology symptom (2). FNDs cause significant disability (3) and economic burden on the health system (4). With usual care, outcomes in patients with FND are often poor (5). They can be improved with modern multidisciplinary treatment, although this requires a good understanding of mechanisms and a consistent approach across the treating team. Thankfully, the increased interest in FND in recent years is helping to improve understanding and management of this heterogeneous disorder. Contemporary FND models posit a mix of factors in what may be considered a “multi-hit” hypothesis – the illness may develop as a complex interplay between genetics, developmental issues, psychosocial and environmental factors, and alterations in neurobiological functions (cognitive, sensorimotor and emotional processing) (6). As such, optimal management of FND should take an integrated approach involving a multidisciplinary team including neurologist, psychiatrist, psychologist, physiotherapist, and on occasions other allied health such as occupational therapists and speech pathologists.

Although FND has traditionally been seen as the illness most at the interface of neurology and psychiatry, it is evident that there is a high rate of co-morbid psychiatric burden associated with it and improvement in mental health is associated with improved outcomes (2). There is little recent published data regarding FND from a psychological or psychiatric perspective. In the 1980's and 1990 attempts were made to psychologically profile FND patients to define the underlying “psychological” cause of the “conversion disorder,” or to explain the illness in terms of personality or psychopathy traits. However, overall, these studies did not identify any significant differences between groups (7). Most recent literature comes from neurology outpatient clinics, and patients are grouped depending on their primary neurological FND symptom. The groups are then analyzed for differences in trauma history and/or psychiatric comorbidities in an attempt to identify aetiological differences between those with dissociative or non-epileptic seizures and those with purely motor symptoms (8–10). Although an association between trauma and FND has been established with estimates ranging from 15 to 77% across studies, attempts to further sub-classify FND based on trauma-type or symptom-type have been mixed (11–13). It is well-known that early childhood trauma is associated with dissociation and the development of dissociative disorders, which would also explain that early trauma plays a role in the development of non-epileptic (dissociative) seizures. While there are many similarities between patients with functional motor symptoms and those with other functional disorders it appears that recent life events, particularly so called “escape” events can be strong triggers for functional motor symptoms (12, 13). There is significant overlap though with many patients having mixed symptoms and trauma histories, making categorization difficult. Anxiety and depression are both reported in 20–40% of patients with FND (14–16). The hope is that a better understanding of underlying factors contributing to the development and maintenance of FND symptoms, will enable a more tailored and effective treatment of these patients in the future. From a mental health point of view, being able to sub-categorize FND patients into psychological/psychiatric groups may identify potential variation in treatment approach, modality and outcomes, helping to make a more individualized treatment plan within the context of the multidisciplinary setting. For example, management of an anxiety disorder differs to the management of post-traumatic stress disorder or personality disorder.

A public multidisciplinary FND clinic was established at Brisbane Mater Adult Hospital in 2015 and ran for ~3.5 years. All patients referred to this clinic were initially assessed by a neurologist for confirmation of FND diagnosis, and then referred for multidisciplinary assessment and treatment by a neuropsychologist and usually a physiotherapist. In some cases, if more severe or comorbid mental illness was suspected, patients were also referred to a neuropsychiatrist. Many of the patients within this cohort were followed up many times, over months and years in some cases, meaning that the authors (who were also the clinicians) knew the patients well. Psychiatric diagnoses were “real-world” clinical diagnoses by experienced neuropsychiatrists and neuropsychologists, based on the Diagnostic & Statistical Manual of Mental Disorders (DSM-V) criteria, as is usual in Australian psychiatric practice.

As part of a retrospective audit of patients attending our FND clinic, we were interested in exploring patient characteristics and underlying psychological conditions that may be contributing to FND symptom presentation. We decided to focus primarily on patients with either motor or dissociation as their primary presenting functional symptom, as these patients are the overwhelming majority within the clinical presentations. Anecdotally, within the cohort of FND patients who were referred to neuropsychiatry or neuropsychology, we observed that patients tended to be highly anxious, or have histories consistent with significant trauma, more so than in the general population. We hypothesized that, consistent with the literature, the incidence of childhood trauma/abuse would be more frequent within the group of FND patients with dissociative symptoms. We also hypothesized that anxiety would be more likely in patients with motor symptoms as their primary FND symptom. In patients with no identifiable psychological or psychiatric comorbidity, we hypothesized that there may be a higher proportion of underlying organic neurological conditions present. We were also interested to explore the prevalence of neurodevelopmental disorders in this FND cohort, as we perceived higher rates of patients with a history of Autism Spectrum Disorder (ASD)intellectual impairment or Attention-Deficit Hyperactivity Disorder (ADHD) in the FND-psychiatry clinic compared to general psychiatry clinics.

## Methods

### Study Design

This exploratory retrospective audit was conducted at the Mater Hospital in Brisbane, Australia. This project received exemption from ethical review from the local human research ethics committee [reference EXMT/MML/61540 (V4)], and additional patient consent was not required. A list of all clinic appointments scheduled for the neurologist at the Neurosciences Functional Neurological Disorder Clinic from May 2015 until June 2019 was compiled.

### Data Collection

The authors performed a retrospective chart review and determined variables of interest. As such, data obtained from records included any mental health diagnoses that were given after assessment by either a neuropsychiatrist or clinical neuropsychologists. Collected data included; demographic information such as age and sex, primary FND symptoms, other neurological and neurodevelopmental disorders, mental health diagnoses, any history of trauma or traumatic experience such as sexual abuse or assault, or (perceived) threat to life from e.g., workplace or traffic accidents (separated out as having occurred in childhood or adulthood, or both), referral to psychiatry, neuropsychology, and physiotherapy. Primary FND symptoms were used to divide the sample into four groups: dissociative (i.e., non-epileptic seizures), motor (i.e., tics, dystonia, tremor, paralysis and weakness), somatoform (i.e., sensory alterations, headache, pain, fatigue) and mixed (i.e., a combination of the aforementioned with no clearly identifiable primary symptom) symptoms. A determination was made based on clinical judgment by the neuropsychologist or neuropsychiatrist, which (if any) psychological factors contributed most strongly to the FND symptom presentation. This was feasible as the researchers were the same clinicians who worked with the patients in the FND clinic and knew the patients well. Identified factors included: anxiety, trauma, anxiety and trauma, depression, depression and anxiety, psychosis, bipolar and no clear psychological factor. The patient list was divided between four clinicians who reviewed each listed patient's medical record and extracted the relevant data recording it into an excel file developed for this purpose. The recorded data were compiled into a master list and recoded from descriptive to numerical variables to allow data analysis. Patients who did not attend an initial appointment with the FND neurologist, or who did not receive a diagnosis of FND, were excluded from the data set.

### Statistics

Statistical analyses were performed using IBM SPSS. Descriptive statistics including means, standard deviations, frequencies and percentages were calculated and reported as appropriate. Differences in age between the primary symptom groups (dissociation, motor, somatoform, and mixed groups) were analyzed using ANOVA. The statistical significance of between group differences in categorical variables were tested using the Chi-square test. All tests were two-tailed with a significance level of *p* < 0.05.

## Results

A total of 316 patients were seen in the clinic. Twenty-eight of these were excluded from further analysis as they were referred to external psychologists and psychiatrists and no information regarding psychological functioning was available, leaving a total of 288 patients. Of these patients, 66.3% were females and 33.7% were males. The age ranged from 15 to 77 years (*M* = 39.43, *SD* = 13.63). 83.7% (*n* = 241) of patients were referred to psychology, 41% (*n* = 118) were referred to psychiatry and 41.3% (*n* = 119) were referred to physiotherapy.

**Figure 1:**
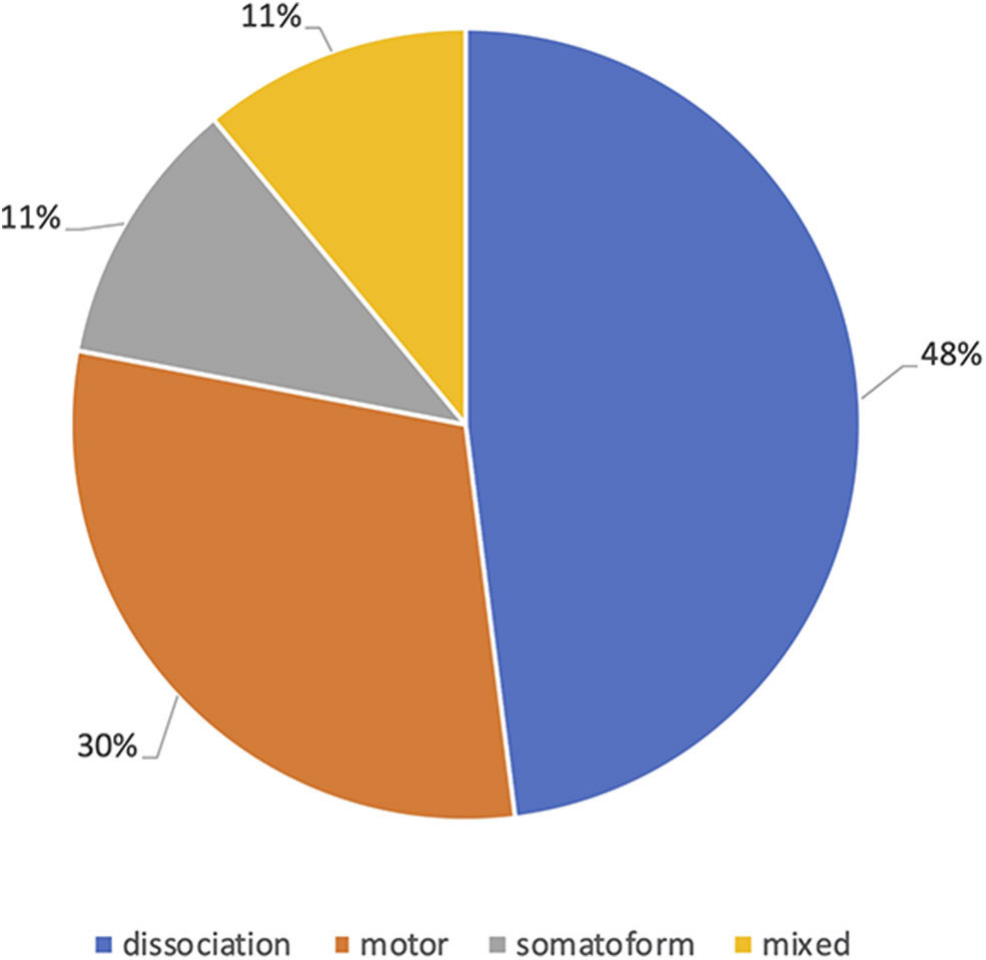
Primary Functional symptoms.

Almost half of the patients seen had dissociation as the primary symptom (48.3%), 29.5% had motor symptoms, 10.8% had somatoform symptoms and 11.5% had mixed symptoms as their primary presenting symptom (Figure 1). There was no difference in the distribution of sex between the four symptom groups. Mean age ranged from 37.21 years (*SD* = 14.01; dissociation group) to 43.45 (*SD* = 11.16; somatoform) years, with only the difference between these groups significant [*F*_(3,284)_ = 2.794, *p* = 0.041, η*p*^2^= 0.029].

Of the total sample, 35.1% had other diagnosed neurological conditions (including epilepsy, acquired brain injury, neoplasms, spinal injury) and 9% had a neurodevelopmental condition (such as intellectual impairment, ASD or ADHD). At least 38.6% of those with other diagnosed neurological conditions had a past and/or current diagnosis of epilepsy. This included patients with a history of juvenile epilepsy, and those with comorbid epilepsy and dissociative symptoms. 73.3% of patients had a current mental health condition.

Of the total sample, 47.9% had a trauma history. A childhood history of trauma was present in 29.9% of cases, whereas adult trauma was present in 18.1% of cases. 52.1% had no reported trauma history. 51.1% of those with dissociation as the primary symptom had a trauma history. In those with a primary motor symptom, 37.6% had a history of trauma, and 58.1% and 51.5% of those with primary somatoform or mixed presentations had a trauma history. A childhood trauma history was present in 35.3% of those who had dissociation as their primary symptom, in 18.8% of those with a primary motor symptom, and in 35.5 and 30.3% of those with primary somatoform or mixed presentations, respectively.

With respect to the psychological factors contributing to the currently presenting FND symptoms, anxiety was judged to be the main contributing factor for 38.2% of patients, trauma 33.0%, trauma and anxiety 2.4%, depression 4.9%, depression and anxiety 4.9%, Bipolar 0.7%, and psychosis 1.0%. In 14.9 % of cases no clear mental health diagnosis was identified.

### Dissociation vs. Motor Symptom Groups

Given that our interest was primarily in the comparison with those who had dissociation or motor symptoms, and that the cell sizes for somatoform and mixed groups were too small for statistical comparisons, the following results include only the contrasts between these two groups.

Table 1 shows the underlying psychological factors in the groups with primary dissociation vs. motor symptoms. Patients with dissociation as their primary symptom were significantly more likely to have current severe psychological diagnoses compared to those with primary motor symptoms χ^2^ (2, *n* = 224) = 12.249 *p* = 0.002. There was no significant difference regarding current anxiety in patients with primary motor symptoms (45.9%) or dissociation (38.1%), *p* = 0.266 or overall trauma history (37.6% in the motor group and 51.1% in the dissociation group, *p* = 0.054). The dissociation group was significantly more likely to have a childhood trauma history (35.3%) than the primary motor group (18.8%), χ^2^ (1, *n* = 224) = 6.911, *p* = 0.010. Compared with the motor symptom group the dissociation group had significantly more patients with other neurological and neurodevelopmental conditions (44.6 vs. 23.5%), χ^2^ (1, *n* = 224) = 10.095, *p* = 0.002. Those that did not have an identifiable psychological factor contributing to their dissociation and motor symptoms, did not differ on whether they had another neurological condition, *p* = 0.109. No clear contributing psychological factors were identified for 12.9% of those with dissociation and 20% of those with motor symptoms. This difference was not significant, *p* = 0.158.

**Table 1:** Percentages of current psychological factors associated with FND in patients with primary dissociation or motor symptoms.

	**No clear psychological factor**	**Anxiety**	**Trauma**	**Depression**	**Bipolar**	**Psychosis**	**Trauma and anxiety**	**Depression and anxiety**
Dissociation *n* = 139	12.9	38.1	37.4	4.3	0.7	1.4	2.9	2.2
Motor *n* = 85	20	45.9	17.6	4.7	1.2	1.2	2.4	7.1

Significantly fewer patients with primary motor symptoms were referred to psychiatry compared to those with primary dissociative symptoms, χ^2^ (1, *n* = 224) = 18.255, *p* = < 0.001. There was no significant difference between patients with dissociation or motor symptoms in terms of whether they were referred to psychology, *p* = 0.208.

## Discussion

While this audit of patients presenting to a tertiary referral Functional Neurological Disorder outpatient service was initiated to assess the characteristics of patients who primarily presented with motor and dissociative functional symptoms, at a broader level there are a number of observations of interest in respect of the data collected.

A large dataset of 288 such patients collected over a 3.5-year period is unusual and has the potential to provide valuable insights into this condition. The larger proportion of females (66.3%) compared to males (33.7%) is consistent with the published literature. What is not consistent with worldwide literature is that nearly half (48.3%) of our sample had dissociation as the primary symptom. The high representation of dissociative attacks may reflect a referral bias in that the Mater Hospital houses an advanced epilepsy service with video-EEG monitoring, and a significant number of referrals to the FND service were patients previously diagnosed with epilepsy where video-EEG capturing of these events had proven that the episodes were non-epileptic in nature.

Our clinical impression that patients with primary dissociative functional symptoms were strongly associated with past trauma is supported by our findings. In the dissociative group of patients, half (51.1%) had a history of trauma. The statistically significant finding of the primary dissociative group having an increased rate of childhood trauma history compared to the primary motor group (37.6%) lends further strength to this association. This is also significant as the Australian population rate of childhood trauma/abuse is estimated at 13%, showing a significant increase in FND compared to the general population (17). The original Freudian idea of conversion as a protective function of the brain against overwhelming unacceptable trauma fits neatly with this group of patients and is compatible with modern neuroscientific principles of a spectrum of neurobiological and psychologically learnt inability of mental representation, sometimes referred to as alexithymia (18).

We did not find statistical support for our second hypothesis that in those patients who presented with primary motor symptoms, anxiety would be a significant association. However, a surprising finding from our dataset was the comparatively higher rate of anxiety (38.2%) in our population compared to depression (4.9%), given that the latter is most often reported at high levels in functional neurological samples in the published literature. One potential explanation is that as investigators we were interested in looking at anxiety as a phenomenon given that it was one of our variables of interest in functional neurological patients, and we were more likely to code anxiety in mixed anxiety and depressive states. An alternative explanation is that anxiety is the primary substrate in a group of functional neurological disorder patients, and a secondary depression is common, but not the primary feature of such patients. In our experience it is rare to see core melancholic depression in functional neurological disorder patients. Previous worldwide studies may have been influenced by the classificatory systems in psychiatry, where if anxiety and depression co-exist, the depressive syndrome is assumed to be dominant and is coded, and the use of screening questionnaires may be biased toward identifying depressive symptoms (19, 20). These issues are intriguing, and perhaps point to anxiety as an important marker for further studies.

When we eliminated all psychiatric and psychological comorbidity, there remained a significant group of functional neurological disorder patients (14.9%) who were classified as “no clear mental health diagnosis.” This may accord with clinical experience of a group of patients, where neither trauma nor anxiety or depressive issues accompany the clinical picture. Perhaps some members of this group are highly alexithymic, and somatise their psychological distress. The emerging neuroscience literature also points intriguingly at a more “neurobiological” group, where higher level dysfunction in the complex circuitry of the brain results in a network “software” problem, without any structural abnormality in the system (18, 21, 22), suggesting that Functional Neurological Disorders represent disorders of networks implicated in volition, emotion and motor control. Neurobiological and neurodevelopmental abnormalities are hypothesized to include an altered sense of self-agency affecting sense of voluntariness of movement, loss of “intentional binding” or the subjective sense of movement and its sensory consequences, as well as bias in evaluating prior information (the Bayesian prior) modifying internal predictions about the subject's world especially in terms of motor control (21). It has also been postulated that prior physical trauma changes the coupling of the amygdala and insula to the motor cortex (23) providing a model neurodevelopmental endophenotype of a brain network that is prone to functional dysconnectivity. There was certainly a high proportion (9%) of patients across the whole cohort of 288 who had a comorbid neurodevelopmental disorder (ADHD, ASD or intellectual impairment). This is a higher rate than in the general Australian population, where rates of ASD are 0.7% and intellectual disability 3% (24, 25). We found that again, the dissociative group had a significantly increased rate of coexisting neurological and neurodevelopmental diagnoses (44.6%) compared to the motor group (23.5%). The underlying “vulnerable brain” idea making these patients more prone to functional symptoms, especially dissociative events, requires further investigation. However, we did not find, as hypothesized, any increased incidence of co-existing organic neurological disease or neurodevelopmental disorders in the group of patients who had no coded psychiatric or psychological morbidity.

There are a number of limitations to this study. This was a retrospective analysis from a single center in Australia. Also, this patient cohort is from a specialist FND clinic from a centre with a large epilepsy unit, which introduces a bias regarding the type of patients being referred to the service. Both these factors limit the generalisability of this study. The FND diagnoses and mental health assessments were made by expert clinicians with many years of experience in the field of Functional Neurological Disorders, but we did not use rating scales in determining psychological drivers. Lastly certain difficulties (and likely inaccuracies) of comorbid neurological diagnoses should be pointed out: these diagnoses were largely derived from hospital records and there is likely significant variability in their accuracy. Taking the diagnosis of underlying epilepsy as an example, some of these diagnoses were determined with video-EEG recording and are very accurate, but the rate we determined in this study included a small number of patients whose current symptoms were determined to be of a dissociative rather than epileptic nature, raising the question whether their previous diagnosis of epilepsy had been accurate. Other patients had an “historical” diagnosis of epilepsy that was made solely on clinical grounds at some point in the past, and there may be a significant misdiagnosis rate in these patients.

In summary, our audit raises interesting questions for further investigation. Can psychological profiles of patients with functional neurological disorder be divided up into an “anxious substrate” cluster, a “trauma dissociative” cluster, a neurodevelopmentally vulnerable group as well as a “neurobiological” group? The elucidation of such profiles holds promise in terms of sub-stratification of the functional neurological disorder syndrome and more targeted treatment approaches for the benefit of this spectrum of patients.

## Data Availability Statement

The raw data supporting the conclusions of this article will be made available by the authors, without undue reservation.

## Ethics Statement

The studies involving human participants were reviewed and approved by Mater Misericordiae Ltd Human Research Ethics Committee. Written informed consent for participation was not required for this study in accordance with the national legislation and the institutional requirements.

## Author Contributions

PP, JF, MB, RD, and AL: conception and drafting of manuscript as well as acquisition of data. All authors contributed to the article and approved the submitted version.

## Conflict of Interest

The authors declare that the research was conducted in the absence of any commercial or financial relationships that could be construed as a potential conflict of interest.
